# Efficacy of Osteopathic Manipulative Treatment for Pain Reduction in Patients With Patellofemoral Pain Syndrome: A Meta-Analysis of Randomized Controlled Trials

**DOI:** 10.7759/cureus.59439

**Published:** 2024-05-01

**Authors:** Blake E Delgadillo, Audrey Bui, Alyssa M Debski, Brooke Miller, Shan Shan Wu, DO

**Affiliations:** 1 Orthopaedic Surgery, Lake Erie College of Osteopathic Medicine, Bradenton, USA; 2 Medicine, Lake Erie College of Osteopathic Medicine, Bradenton, USA; 3 Anatomy, Lake Erie College of Osteopathic Medicine, Bradenton, USA; 4 Urology, Lake Erie College of Osteopathic Medicine, Bradenton, USA; 5 Allergy/Immunology, Allergy/Immunology Associates, Inc., Mayfield Heights, USA

**Keywords:** orthopedics, orthopedic sports medicine, randomized controlled trial (rct), a meta-analysis, manual-therapy, doctor of osteopathic medicine, osteopathic manipulative medicine (omm), osteopathic manipulation, osteopathic manipulative treatment (omt), patellofemoral pain syndrome

## Abstract

Patellofemoral pain syndrome (PFPS) is among the most common causes of musculoskeletal pain in the United States. It is defined as retropatellar or peripatellar pain that is reproduced with functional activities that load the patellofemoral joint in a flexed position, such as stair climbing or squatting. While it presents in both adolescents and adults, it is commonly found in physically active individuals, such as athletes and military recruits. Exploring the role of osteopathic manipulative treatment (OMT) in PFPS is of particular interest given the absence of a definitive treatment and the poor long-term prognosis associated with PFPS. This meta-analysis includes three studies exploring the use of OMT to reduce pain in patients suffering from PFPS and exploring the efficacy of OMT as a primary intervention. In these studies, pain assessments, pre-treatment, and post-treatment follow-up of at least 30 days were performed using a 10-cm visual analog scale (VAS). The mean difference in pain between OMT and no treatment (NT) groups using the random effects model was -3.95 (-6.39; -1.50) with a p<0.01, suggesting OMT resulted in significant knee pain reduction in those with PFPS. A measure of heterogeneity, known as I^2^, was found to be high at 97%, which suggests caution should be taken when interpreting the overall results. Given the lack of definitive treatment and the poor long-term prognosis for PFPS, the authors suggest OMT provides an effective option for pain relief in patients with PFPS. Further research is needed to provide results that may be more clinically applicable or valuably interpreted.

## Introduction and background

Patellofemoral pain syndrome (PFPS) is a condition characterized by pain around or behind the patella. Approximately 15%-45% of individuals are affected by PFPS, making it stand out as one of the most common types of knee pain [1]. This condition is more prevalent among adolescents (28.9%) and adults (22.7%) who are actively engaged in physical activities, such as athletes and military recruits [2]. Additionally, it is observed that females are 2.23 times more likely to have PFPS than males [3]. The etiology of PFPS involves several factors, such as vastus medialis atrophy, lower limb muscular imbalance, overuse of lateral structures, and delayed core muscle activity [4-6]. Furthermore, biomechanical issues beyond the knee joint, such as in the trunk and hip, have been hypothesized to influence the development of PFPS [7, 8], thus illustrating the complexity of multifactorial interactions that contribute to the development of this condition. 

The anterior knee pain associated with PFPS becomes more pronounced when bearing weight on a flexed knee [9] and typically increases during prolonged periods of sitting or when descending stairs [10]. Symptoms of PFPS are frequently recurrent and longstanding, lasting for several years. These symptoms can become severe enough to lead to inactivity, withdrawal from sports, and reduced work activity [2]. Regarding long-term prognosis, the condition is often associated with a significant level of disability, which can be debilitating and persistent [2]. 

Treatment for PFPS is typically conservative, with the primary goals being to reduce pain, improve patellar alignment, and facilitate a return to the prior functional level [9]. Non-operative approaches include medially directed tape application, nonsteroidal anti-inflammatory drugs, and exercise regimens targeting the trunk, hip, and decreased extremity muscle mass [11]. Surgical options involve arthroscopy, lateral release, and medial tissue tightening, though these have been deemed ineffective [12,13]. Current literature suggests that a majority of patients may see improvement through the use of conservative treatments [9]. However, studies focused on the long-term outcomes of PFPS have reported that up to 57% of patients continue to be affected over five to eight years [14,15]. 

For those with PFPS, studies have shown that osteopathic manipulative treatment (OMT) can lead to significant pain reduction and increased functionality [16]. Osteopathic manipulative treatment specializes in restoring proper biomechanical forces throughout the body to relieve abnormal knee stress, making use of techniques aimed at correcting joint and myofascial dysfunctions [17]. The efficacy of OMT has been well documented in the management of other chronic conditions, such as low back pain, chronic knee osteoarthritis, and headache [18-20]. To date, there has not been a comprehensive systematic review specifically elucidating the role of OMT in PFPS management. Given this, our meta-analysis aims to evaluate the effectiveness of OMT in reducing knee pain among individuals with PFPS. In our analysis, we compared the pain relief achieved by OMT against no treatment. Our inclusion criteria focused on randomized controlled trials (RCTs) that involved males and females in their 20s and 30s with PFPS and assessed pain using a visual analog scale (VAS). We excluded non-English studies, non-randomized trials, and research published before 2000, among other criteria. 

## Review

Materials and methods

Research Question

Does OMT cause a more significant reduction in pain than no treatment in patients with patellofemoral pain syndrome? 

Inclusion Criteria 

Population: Male or females in their 20s or 30s with a body mass index (BMI) classified as normal (18.5-24.9 kg/m^2)^ or overweight (25-29.9 kg/m^2^) with a diagnosis of PFPS, defined as anterior or retropatellar pain provoked by two or more of the following activities: prolonged sitting, stair ascent, stair descent, compression, squatting, kneeling, or isometric quadriceps contraction; Study design: Randomized controlled clinical trials; Intervention: Osteopathic manipulative treatment versus no treatment (NT) for the treatment of PFPS; Comparison: Studies with an active control group; Outcome: The primary outcome was measured by focusing on pain on a 10-cm VAS before and after OMT at a follow-up of at least 30 days.

Exclusion Criteria 

Non-relevance: Manuscripts unrelated to OMT for PFPS; Non-randomized controlled clinical trials: Case reports, cross-sectional, prospective or retrospective cohort, non-randomized trials, qualitative or systematic reviews; Language: Studies available in only non-English; Publication date: Published before the year 2000; Outcome: Treatment not defined as osteopathic, failure to report pain on VAS, and lack of follow-up of at least 30 days; Comorbidities: prior spine or knee surgery, competing knee conditions, lumbosacral pathology, or any neurologic disease 

Search Strategy

**Figure 1:**
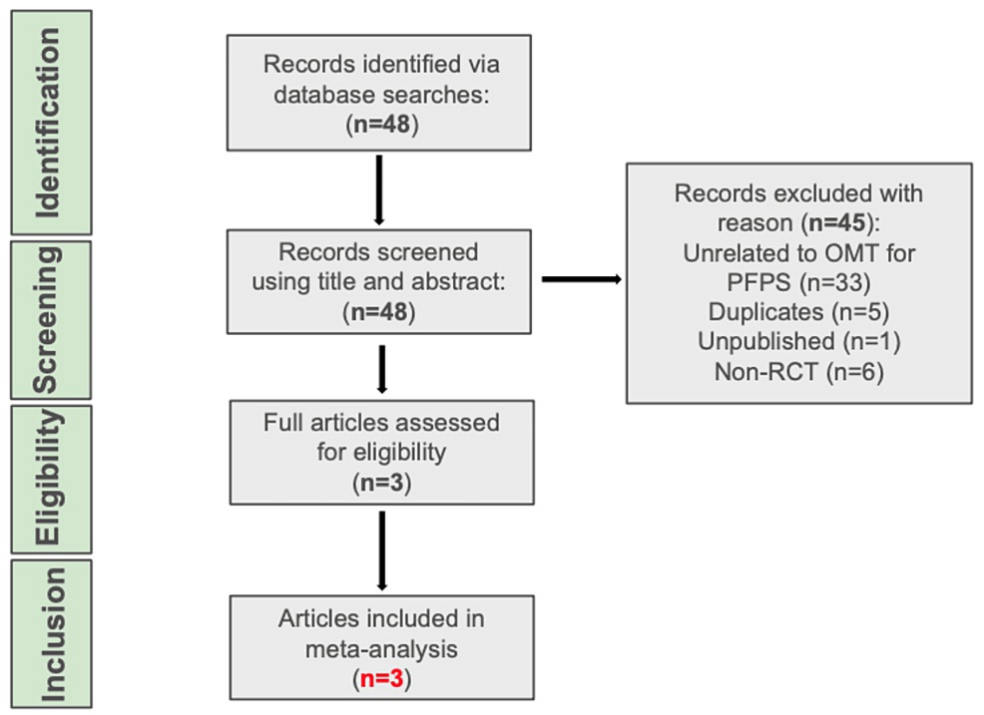
A PRISMA flowchart depicting the literature search and selection process n: number; OMT: osteopathic manipulative treatment; PFPS: patellofemoral pain syndrome; RCT: randomized controlled trial; PRISMA: Preferred Reporting Items for Systematic Reviews and Meta-Analyses

The Preferred Reporting Items for Systematic Reviews and Meta-Analyses (PRISMA) criteria (Figure 1) was employed for article selection [21]. An extensive electronic database search was conducted on titles up to November 2023 in the following databases: PubMed, ScienceDirect, OSTMED.DR, Osteopathic Research Web, OSTLIB, Osteoevidence, and American Academy of Osteopathy Journal. Search terms included “patellofemoral pain syndrome”, “PFPS”, “patellofemoral pain” or “PFP” with “randomized controlled trial”, “RCT”, “osteopathy”, “osteopath”, “osteopathic principles & practice”, “OPP”, “osteopathic manipulative medicine”, “OMM”, “osteopathic manipulation”, “osteopathic manipulative treatment”, “osteopathic manipulative therapy”, “osteopathic treatment”, “osteopathic technique”, “OMT”, and “OMTh”. 

Data Sources 

Manual searches of relevant systematic reviews and meta-analyses were also conducted to identify other studies not listed under our database searches. 

Study Selection

Duplicates were recognized and were not included in the statistical analysis more than once. Non-RCTs and studies unrelated to patellofemoral pain syndrome or osteopathic manipulative treatment were not included. There were no disagreements between reviewers. 

Data Extraction

Data related to study design, sample characteristics, sample size, average rating on a 10-cm VAS, standard deviation, and confidence intervals were extracted from each study. Confidence intervals were calculated for studies that did not report such numbers. 

Quality Assessment

Only RCTs were included to minimize bias within each study. Bias was assessed using statistical analysis. There were no disagreements between reviewers (AB and BD). 

Statistical Analysis

All analyses and graphs were run and produced in R Statistical Software (v4.1.2; R Core Team 2021) using the package meta version 6.5-0. The meta-analytical method for all analyses used the inverse variance method with a restricted maximum-likelihood estimator for τ2. Additionally, the Q-profile method was used for confidence intervals of τ2 and τ. The risk of bias assessment was run in Microsoft Excel (Microsoft Corp., Redmond, WA) using the Cochrane Risk of Bias Assessment Tool version 2 by two reviewers (AB and BD) [22].

Publication Bias

**Figure 2:**
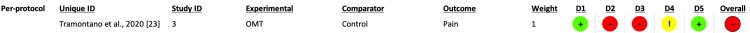
Risk of bias for the per-protocol study using the Cochrane Risk of Bias Assessment Tool version 2 Independently completed by two reviewers (AB and BD) using the Cochrane Risk of Bias Assessment Tool version 2, as described by Sterne et al. [24]. Per-protocol study: Tramontano et al. [23] D1: randomization process; D2: deviations from the intended interventions; D3: missing outcome data; D4: measurement of the outcome; D5: selection of the reported result; +: low risk; !: some concerns; -: high risk

**Figure 3:**
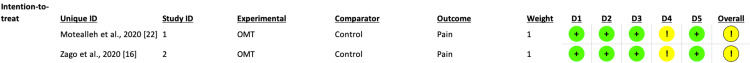
Risk of bias for the intention-to-treat studies using the Cochrane Risk of Bias Assessment Tool version 2 Independently completed by two reviewers (AB and BD) using the Cochrane Risk of Bias Assessment Tool Version 2, as described in Sterne et al. [24]. Intention-to-treat studies: Motealleh et al. and Zago et al. [16, 22] D1: randomization process; D2: deviations from the intended interventions; D3: missing outcome data; D4: measurement of the outcome; D5: selection of the reported result; +: low risk; !: some concerns; -: high risk

It is possible publication bias limited our search as only specific studies focused on OMT were included that were related to the reduction of pain using a VAS at a follow-up of at least 30 days in patients diagnosed with PFPS. For each study, the Cochrane Risk of Bias Assessment Tool version 2 was used to assess the bias in this manuscript (Figures 2, 3) [16,22-24].

Results 

Literature Search 

The total number of studies found was 14. After the removal of duplicates, articles unrelated to OMT for PFPS, non-RCTs, and unpublished articles, three articles were assessed and subsequently included (Figure 1) [16,22,23]. 

Characteristics of Studies Included 

Three studies [16,22,23] were included in this meta-analysis, and the characteristics of each can be found in Table 1. 

**Table 1:** Description of studies included in statistical analysis RCT: randomized control trial; PFPS: patellofemoral pain syndrome; HVLA: high-velocity low-amplitude; AFR/MFR: articular/myofascial release; BLT: balanced ligamentous tension; VM: visceral manipulation; CM: cranial manipulation; *: diaphragmatic region, pelvic floor region, lumbar/abdominal lateral wall region; VAS: visual analog scale

Study		Total sample size (N)	Design	Diagnosis	Osteopathic manipulative treatment	Anatomic region manipulated		Pain scale measured
Motealleh et al., 2020 [22]		44	RCT	PFPS	HVLA	Lumbopelvic		VAS
Tramontano et al., 2020 [23]		35	RCT	PFPS	AFR/MFR, BLT, VM, CM	Head, cervical, thoracic, lumbar, sacral, pelvic, lower extremities, upper extremities, ribs, abdomen, and other regions*		VAS
Zago et al., 2020 [16]		54	RCT	PFPS	HVLA, MFR	Lumbosacral spine and/or hip, sacroiliac joint, knee, and ankle, lumbar square muscle and/or fascia lata tensor, iliopsoas, piriformis, quadriceps, and gastrocnemius muscles		VAS

Risk of Bias Assessment 

The risk of bias was assessed using the Cochrane Risk of Bias Assessment Tool version 2 [24]. The risk of bias in the intention-to-treat trials was low, with some concerns (Figure 3). In the per-protocol study, the risk of bias was found to be high (Figure 2). 

Findings 

**Figure 4:**
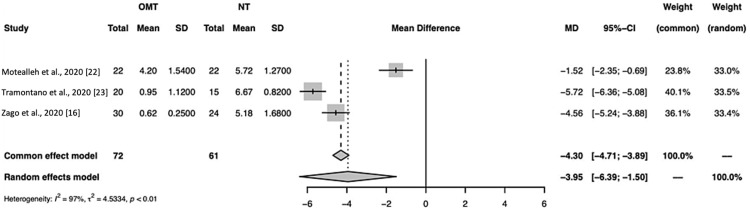
A forest plot of pain assessment using a visual analog scale compares osteopathic manipulative treatment versus no treatment for all studies included. OMT: osteopathic manipulative treatment; NT: no treatment; SD: standard deviation; CI: confidence interval Studies included: Motealleh et al., Tramontano et al., and Zago et al. [16,22,23]

The results for common and random effect models in this manuscript can be found in Figure 4. 

In this meta-analysis (Figures 1, 4), the total number of studies included was three, and the total number of observations was 133 (OMT = 72, NT = 61). Tramontano et al. had the highest effect size, which indicates the magnitude of the reduction in pain due to OMT was the highest (mean difference = 5.72) [23]. Overall, the effect sizes were observed to be considerably different, suggesting overall study heterogeneity, which is the extent to which effect sizes vary within the meta-analysis (Figure 4). The size of the box is proportional to its weight, which was highest for Tramontano et al. in both the random (33.5%) and common (40.1%) effect models [23]. Tramontano et al. had the highest precision, which is derived from the confidence interval range being the smallest (-6.36; -5.08) [23]. The mean difference between OMT and NT groups, respectively, for the common effect model was -4.30 (-4.71; -3.89) and for the random effects model was -3.95 [-6.39; -1.50] with a p<0.01. This suggests the reduction in pain due to OMT compared to NT was significant. The percentage of variation across studies due to heterogeneity (I^2^) was 97% (Figure 4). According to the Higgins and Green study, heterogeneity between 50% and 90% represents substantial heterogeneity, and 75%-100% represents considerable heterogeneity [25]. 

**Figure 5:**
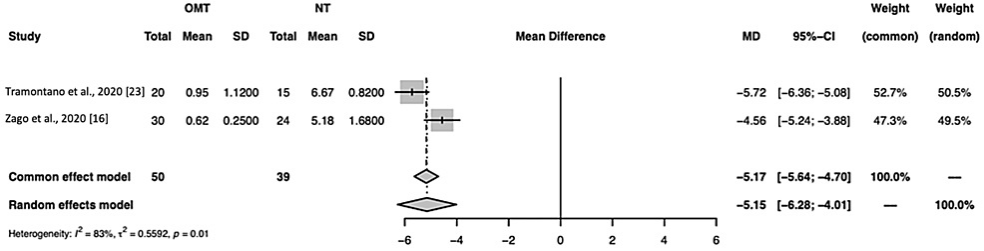
A forest plot of pain assessment using a visual analog scale compares osteopathic manipulative treatment versus no treatment for studies focused on generalized body manipulation. OMT: osteopathic manipulative treatment; NT: no treatment; SD: standard deviation; CI: confidence interval Studies focused on generalized body manipulation: Tramontano et al. and Zago et al. [16,23]

In Figure 5, a meta-analysis run for Tramontano et al. and Zago et al. computed the mean difference for the common and random effect models as -5.17 (-5.64; -4.70) and -5.15 (-6.28; -4.01), respectively, with p = 0.01 [16, 23]. These data suggest the decrease in pain due to OMT was significant. The I^2^ of this analysis was 83%, which is slightly lower than the I^2^ for the analysis including all three studies (I^2^ = 97%) (Figure 5).

**Figure 6:**
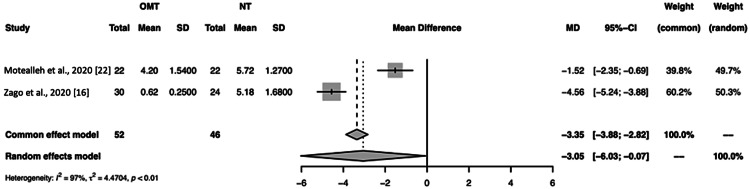
A forest plot of pain assessment using a visual analog scale compares osteopathic manipulative treatment versus no treatment for intention-to-treat studies. OMT: osteopathic manipulative treatment; NT: no treatment; SD: standard deviation; CI: confidence interval Intention-to-treat studies: Motealleh et al. and Zago et al. [16, 22]

Figure 6, which compares Motealleh et al. and Zago et al., the mean difference was -3.35 (-3.88; -2.82) and -3.05 (-6.03; -0.07) for the common and random effect models, respectively, with a p<0.01 [16,22]. This, like the other analyses, suggests that pain reduction due to OMT versus NT was significant, although the I^2^ was considerably high at 97%.

**Figure 7:**
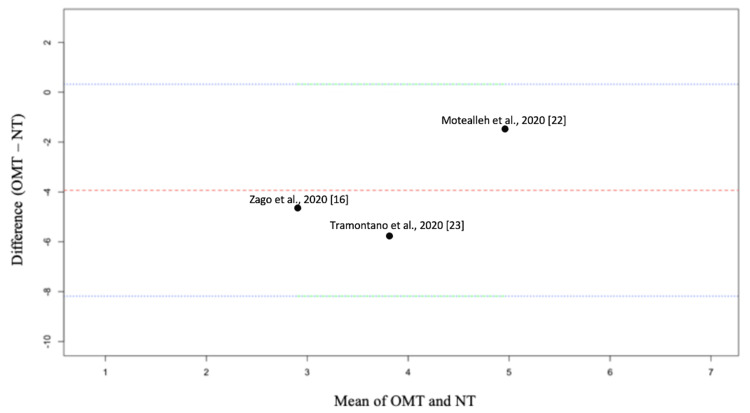
Bland Altman plot for pain measured using a visual analog scale at a follow-up of at least 30 days after osteopathic manipulative treatment versus no treatment. OMT: osteopathic manipulative treatment; NT: no treatment; upper blue/green line, the upper limit of agreement; lower blue/green line, the lower limit of agreement; middle red line, bias; dots labeled with each study, the difference between OMT versus NT [16,22,23]

The data in the Bland Altman plot, which is a plot used as a visual for the difference between VAS after OMT/NT plotted against the average VAS between OMT/NT, reveal the mean difference between VAS for OMT versus NT for the included studies is within the limits of agreement (Figure 7). This suggests the difference between VAS measured for OMT and VAS measured for NT is consistent across the range of measurements. In other words, the VAS used for each method is in agreement, suggesting the method of measuring pain after OMT or NT is a suitable measurement tool.

Discussion 

This meta-analysis of three studies assessed the effectiveness of OMT compared to no treatment in reducing knee pain in patients with PFPS. Various OMT techniques were employed, which consisted of high-velocity, low-amplitude (HVLA), articular/myofascial release (AFR/MFR), balanced ligamentous tension (BLT), visceral manipulation (VM), and cranial manipulation (CM) (Table 1). Areas treated included head, cervical, thoracic, lumbar, sacral, pelvis, upper extremity, ribs, abdomen, sacroiliac, joint, knee, ankle, lumbar square muscle, tensor fascia lata, iliopsoas, piriformis, quadriceps, and gastrocnemius (Table 1). The primary outcome measured was the magnitude of pain reduction using a VAS, which was used by researchers to assess pain at a follow-up of at least 30 days. 

The results from our meta-analysis including all three studies showed a significant reduction in knee pain in patients with PFPS receiving OMT, with an average VAS pain score being 3.95 points lower in the OMT treatment group compared to the control group (p<0.01; Figure 4). However, substantial heterogeneity was observed (I^2^ = 97%), potentially due to a small sample size and differing interventions. When considering the I^2^ value, the large value suggests caution should be taken when interpreting the overall results. This is due to a large I^2 ^representing almost all of the variability in effect sizes due to heterogeneity. Of note, each study employed various methods and dosages of OMT. The consistency across the groups for OMT or NT may not be consistent enough to be clinically applicable or valuably interpreted. Although we present both common and random effect models, due to the aforementioned heterogeneity, the data presented in this discussion were interpreted using the random effects model. It is also important to note that I^2^ estimates themselves should be interpreted with caution for meta-analyses with a limited number of studies [26]. This study found that meta-analyses with less than 500 data points and less than 15 trials, such as ours, tend to experience large variations in I^2^ regardless of true heterogeneity. For this reason, we believe our small sample size resulted in an I^2^ that may have overestimated heterogeneity. 

Overall, this meta-analysis has some concerns about bias due to the bias within the studies included (Figures 2, 3). The overall bias was in large part due to the high bias found in Tramontano et al. (Figure 2) [23]. The bias found in Tramontano et al. stemmed from the obvious differences in physical contact in the placebo treatment given to the control group versus the actual treatment in the intervention group [23]. Hohenschurz-Schmidt, et al. discussed that the difference in physical contact between the groups could have led to a lack of interest in the respective group and, therefore, attrition [27]. They also argued that the physical contact could have been minimal enough to compromise blinding. Additionally, the authors stated that Tramontano et al. did not include the 12.5% of participants that were lost in their analysis [23,27]. Therefore, when assessing bias, a per-protocol approach was used for this study (Figure 2). The study did not perform the proper analyses to address these issues, which could have further compromised their results. Interestingly, the effect size and precision were highest in this study in our meta-analysis, but these values may have been overestimated due to the aforementioned bias. 

We conducted a subanalysis, eliminating Tramontano et al. due to the high risk of bias according to our risk of bias analysis and the assessment by Hohenschurz-Schmidt et al. [23,27]. However, when comparing only Zago et al. and Motealleh et al., the reduction in pain for OMT versus NT remained significant (p<0.01), but the heterogeneity remained high (I^2^ = 97%) (Figure 6) [16,22]. These data suggest that despite the high risk of bias, heterogeneity was virtually unaffected by Tramontano et al. [23]. Additionally, due to the high I^2^, a Bland-Altman plot was used to assess the suitability of the VAS as a measurement tool for pain. As seen in Figure 7, agreement was noted for the VAS used for each study, which suggests this tool was a suitable form of measurement. 

The potential bias and lack of standardization shed light on the imperative need for additional osteopathic research. The rarity of osteopathic research leads to a lack of a solid foundation for researchers to model future studies. Increasing osteopathic studies may create opportunities to more accurately assess the efficacy of osteopathic treatments, and this could, in turn, allow for the improvement of patient outcomes more conservatively. For instance, expanding the current arsenal of pain treatment in PFPS with adjuvant therapy such as OMT may be effective in reducing pain, but more research is needed. In an article by Beverly, the author postulates that part of the reason for the lack of osteopathic research is a lack of available funding to support research projects [28]. It was found that colleges of osteopathic medicine only received 0.1% of all NIH-funded grants in the United States [28]. This could explain the research gap that the field of osteopathy faces compared to allopathic medicine. Furthermore, in an article by Clark and Blazyk, the authors recognize the lack of osteopathic research, suggesting combined osteopathic and allopathic research that could allow for the analysis of OMT effectiveness against select treatments [29]. Nevertheless, evidence-based research builds credibility, and therefore, the lack of current OMT research potentially threatens the reputation of osteopathic practices. This emphasizes the importance of conducting osteopathic research and providing evidence to support this practice. 

We also performed a subanalysis based on the type of OMT performed. The approach of using clinical judgment to locate and treat somatic dysfunctions not constrained to the region of the knee aligns with how a clinician would treat patients in their office. This approach is consistent with at least two models of osteopathic care, particularly the neurologic model and the biomechanical model. The goal of the neurological model is to balance the autonomic system and decrease afferent pain signals, while the biomechanical model aims to restore the mobilization of joints and address restrictions in myofascial and soft tissue [30]. Interventions that focus treatment on a single pathologic structure could lead to poor results based on the theory behind osteopathy [31]. Motealleh et al. specifically used only lumbopelvic HVLA for treatment [22]. Therefore, we compared Tramontano et al. and Zago et al., which performed generalized OMT [16, 23]. As described in Figure 5, Tramontano et al. utilized techniques such as MFR, BLT, VM, and CM on the head, upper body, and lower body, whereas Zago et al. performed HVLA and MFR only on regions of the lower body [16,23]. Interestingly, we found that the average VAS pain score was 5.1 (-6.2, -4.0) points lower in the OMT treatment group compared to the control group (p<0.01, I^2^=83%) (Figure 5). This subanalysis had an absolute mean difference that was 1.2 points greater with a smaller confidence interval compared to the meta-analysis, which included all three studies. This highlights the value of properly implementing osteopathic techniques using the biomechanical and neurological models to increase efficacy in the treatment and further supports the use of OMT treatment for the management of PFPS.

Important limitations of this meta-analysis include minimal data points, a low number of trials, a lack of standardized OMT protocols, overall moderate amounts of bias, and large heterogeneity. We postulate that the high heterogeneity was due to multiple reasons, which may include a low number of data points, a low number of trials, bias, a lack of standardization, and varying types of OMT performed. Due to the aforementioned reasons, generalizability is limited, and clinical application must be interpreted with caution. Although substantial heterogeneity was found, the results for each analysis consistently showed pain was significantly decreased in the OMT groups. Future studies, including standardized OMT treatment and protocols to decrease heterogeneity, may pose a potential solution. Additionally, we recommend increasing sample sizes to add power to the data.

To the best of our knowledge, there are currently no other existing meta-analyses that specifically assess the reduction in knee pain in patients with PFPS after OMT. Despite the existence of research regarding PFPS, there are limited studies that involve OMT, further indicating the need for more osteopathic research. This reinforces the importance of our analysis and its contribution to further understanding the clinical significance of OMT as a potential therapeutic intervention for PFPS. Overall, our analysis suggests a significant reduction in PFPS pain after OMT versus NT. Therefore, we believe that the addition of OMT would be an effective adjuvant therapy to reduce pain for patients with PFPS.

## Conclusions

In this meta-analysis, we investigated three RCTs that compared the reduction of pain in patients with PFPS using a VAS for OMT versus NT at a follow-up of at least 30 days. When comparing all the studies, it was found that OMT provides a significant reduction in pain for patients with PFPS. Our subanalysis, focusing on studies with lower bias (Motealleh et al. and Zago et al.), demonstrated a significant reduction in pain after OMT, despite persistently high heterogeneity. Our subanalysis isolating higher-quality OMT interventions revealed a higher reduction in pain after OMT with smaller confidence intervals and lower heterogeneity. The results of each analysis should be interpreted with caution as the large heterogeneity may affect the clinical applicability and impact the ability to valuably interpret; however, due to the small sample size, number of trials, and number of studies, the heterogeneity was likely overestimated. Overall, the authors suggest that OMT provides an effective option for pain relief in patients with PFPS.
